# Might life-threatening acute pulmonary edema occur after using recombinant tissue plasminogen activator? A case report

**DOI:** 10.1186/s12883-021-02371-w

**Published:** 2021-09-09

**Authors:** Xue-Bing Chen, Dong Qu, Qing Zhang, Xia Yue, Dong-Fang Qiao

**Affiliations:** 1 School of Forensic Medicine, Southern Medical University, 510515 Guangzhou, China; 2 Institute of Legal Medicine, Hannover Medical School, 30625 Hannover, Germany; 3 Nan Fang Hospital Pharmacy Department of the Southern Medical University, 510515 Guangzhou, China

**Keywords:** Recombinant tissue plasminogen activator, Acute ischemic stroke, Acute pulmonary edema, Forensic pathology, Case report

## Abstract

**Background:**

Recombinant tissue plasminogen activator (rt-pa) is the first-line drug for the treatment of acute ischemic stroke, and can lead to some complications.There were rare reports of death due to acute pulmonary edema during rt-pa thrombolysis treatment.

**Case presentation:**

This study reports a 30-year-old man was diagnosed with acute ischemic stroke and underwent rt-pa thrombolytic therapy. Finally he died despite active rescue.

**Conclusions:**

The autopsy revealed that he died of acute pulmonary edema. This case suggests that it is necessary to pay close attention to the changes of vital signs during thrombolysis and be aware of possibility of pulmonary edema during thrombolysis.

## Background

Recombinant tissue plasminogen activator (rt-pa) is the first-line drug for the treatment of acute ischemic stroke [1], but rt-pa can lead to a variety of complications. The most common complications involve bleeding, such as intracranial hemorrhage, gastrointestinal bleeding and genitourinary bleeding, and other complications, such as nausea and vomiting, hypotension and allergic reactions, may occur [2]. However, there were rare reports of death due to acute pulmonary edema during rt-pa thrombolysis treatment. This case suggests that changes of vital signs should be closely observed and the possibility of pulmonary edema during thrombolysis should be considered and corresponding rescue measures should be taken appropriately.

## Case presentation

A 30-year-old male was admitted to the Shenzhen Baoan District Central Hospital due to weakness of the left limbs for 2 h. The patient suddenly experienced weakness and numbness in the left limbs and the inability to stand, accompanied by dizziness, slurred and nonfluent speech, and dysphagia when the neck was massaged 2 h previously. He had a history of hypertension, but the details were unknown. A physical examination on admission revealed the following: pulse (P), 82 times/min; blood pressure (BP), 152/95 mmHg, D-dimer 0.17 mg/L, AST 95U/L,LDH 293U/L. On neurological examination, he was poor spirit; clear consciousness; nonfluent speech; ability to answer questions; equal-sized and round pupils with a diameter of 3.0 mm; sensitivity to light; symmetrical bilateral frontal lines; not significantly shallow of bilateral nasolabial folds; middle placement of the tongue; no edema in the lower limbs. There were weakness of left limbs (muscle strength in the left upper limb weakness of grade 3; muscle strength in the left lower limb weakness of grade 3 with weakened muscle tone; superficial hypoesthesia of the left limbs). there was normal in the right limbs. No bilateral pathological signs. He had a National Institutes of Health Stroke Scale (NIHSS) score of 9. A computerized tomography (CT) examination showed no significant abnormalities. His diagnosis was acute ischemic stroke and hypertension grade 1. The patient had an indication for rt-pa thrombolysis without related contraindications according to the Chinese guidelines (2014 and 2018 and AHA/ASA criteria 2013 and 2018). The patient’s muscle strength in the left upper and lower limbs recovered to grade 5 after thrombolysis for 30 min. The patient was sudden with cough, blood in sputum without a headache, vomiting, vertigo, pallor, chest pain or dyspnea after thrombolysis for 45 min. At 50 min, he experienced chest pain and a heart rate of 167 beats/min, and his blood oxygen saturation decreased rapidly from 99 to 89 %. When rt-pa was infused 53 min later, his blood oxygen saturation decreased to 65 % and he went into cardiac arrest. Despite various rescue measures, the patient eventually died.

**Fig. 1:**
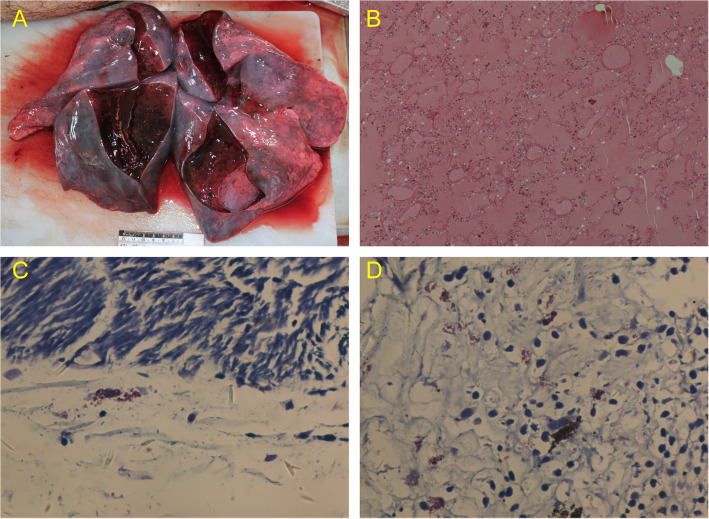
The surfaces of both lungs were dark red with significant congestion and a large amount of foamy hemorrhagic edematous fluid (**A**). HE staining showed significant congestion, diffuse pulmonary edema and local hemorrhage in the bilateral pulmonary stroma (**B**, HE×50). Giemsa staining showed orange-red larynx and hilar mastocytes and the degranulation of some mastocytes in the larynx and hilar (**C**-**D**, Giemisa×100)

An autopsy was performed on the second day after death. The body was 167 cm long. No rashes, hemorrhages or ecchymosis were found on body surface examination. The autopsy mainly found a large amount of bloody edema fluid in the oral, nasal cavity and tracheal cavity with significant mucosa congestion. Both lungs exhibited congestion and edema macroscopically, showing a large amount of foamy bloody edema fluid overflow (Fig. 1A). No significant pathological changes were observed in other organs. Microscopically, numerous arteriolar walls showed thickening and hyalinization, and some wall calcification in the brain. The pulmonary interstitium was hyperemic with diffuse pulmonary edema with local hemorrhage (Fig. 1B). Some of the alveoli showed compensatory emphysema, but the rest of the structures were unremarkable. Submucosal edema was observed in the larynx. Some mast cell degranulation signs were observed in the larynx, hilum, and digestive tract (Fig. 1C-D). The total serum IgE content was 11.55 IU/ml.

The patient died of respiratory and circulatory failure due to acute pulmonary edema caused by rt-pa thrombolysis in acute ischemic stroke.

## Discussion and conclusions

With the continuous promotion and intensive study of intravenous rt-pa thrombolytic therapy, some complications have attracted the attention of clinicians and researchers [2]. However, there were rare cases of death due to acute pulmonary edema without anaphylaxis during rt-pa thrombolysis. This paper reports a case of a young man who developed acute pulmonary edema during rt-pa thrombolysis treatment in one hour and finally died.

Pulmonary edema is mainly divided into cardiogenic and noncardiac causes [3]. The autopsy revealed that there were no fatal pathological changes related to cardiogenic pulmonary edema. Regarding neurogenic pulmonary edema, it seems that there were no findings of insula infarct in CT, but an early insula infarct could not be excluded. Therefore, neurogenic pulmonary edema could not be also excluded for the cause of death. Although the autopsy found hypertension-related signs, there were no complications of hypertension and clinical signs related to an acute attack. The autopsy did not detect manifestations of pulmonary infection and dysplasia. The occurrence of acute pulmonary edema caused by pulmonary disease itself was also excluded. No other special drugs were used simultaneously during rt-pa thrombolysis, therefore the correlation between rt-pa and acute pulmonary edema could not be excluded.

Anaphylaxis can occur during rt-pa treatment and mostly occurs at one hour of thrombolytic therapy [4]. In this case, the patient did not present common allergic reaction symptoms such as rash, pruritus, and orolingual edema. The autopsy did not show a systemic allergic reaction (such as significant laryngeal edema and eosinophil infiltration) or shock-related circulatory failure. Only mild laryngeal edema was found, and some mast cell degranulation signs were observed in the larynx, hilum, and digestive tract. These manifestations did not indicate an allergic reaction in the patient.

It has been shown that rt-pa converted plasminogen then to plasmin *in vivo*, which can activate the complement cascade lead to mast cell degranulation and the release of potent inflammatory mediators, such as histamines, proteases, chemokines, cytokines, and metabolites of arachidonic acid, causing angioedema [5]. In addition, rt-pa-catalyzed plasmin is a protease that breaks down high molecular weight kininogen into bradykinin, which a potent vasorelaxant substance that rapidly promotes vasodilatation and increases vascular permeability, causing angioedema [6]. The distribution half-life and elimination half-life of rt-pa in patients were 3.3–4.8 and 16–88 min, respectively [7]. This time window is consistent with the time of occurrence of acute pulmonary edema in this case. Rt-pa might activate both the complement system and the bradykinin pathway, leading to acute pulmonary edema.

This case suggests that the possibility of pulmonary edema should be aware during thrombolysis and close attention should be paid to the changes of vital signs during thrombolysis so that corresponding rescue measures can be taken.

## Data Availability

Not applicable.

## Abbreviations

rt-pa: Recombinant tissue plasminogen activator
P: Pulse
BP: Blood pressure
NIHSS: National Institutes of Health Stroke Scale
CT: Computerized tomography
